# Disease progression in *IMPDH1* gene-associated rod-cone dystrophy caused by a rare p.Thr244Pro heterozygous variant

**DOI:** 10.1007/s10633-026-10084-z

**Published:** 2026-02-15

**Authors:** Mirella Barboni, Eszter Jávorszky, Mathieu Quinodoz, Ji Hoon Han, Miklós Resch, Zoltán Zsolt Nagy, Carlo Rivolta, Kálmán Tory, Viktória Szabó

**Affiliations:** 1 Department of Ophthalmology, Semmelweis University, Budapest, Hungary; 2 Institute of Molecular and Clinical Ophthalmology, Basel, Switzerland; 3 Pediatric Center, MTA Center of Excellence, Semmelweis University, Budapest, Hungary; 4 Department of Ophthalmology, University of Basel, Basel, Switzerland

**Keywords:** Inherited retinal dystrophy, Autosomal dominant retinitis pigmentosa, *IMPDH1* gene, Mild phenotype, Electroretinography

## Abstract

**Purpose:**

A hallmark of the *IMPDH1*-related rod-cone dystrophy is the highly variable expressivity. Disease onset, severity and progression are variant-dependent due to specific affected photoreceptor mechanisms, underscoring the importance of genotype–phenotype correlations. We provide a detailed clinical characterization of a patient with rod-cone dystrophy caused by a rare *de novo* missense *IMPDH1* variant.

**Case description:**

A 33-year-old female patient was assessed over a period of four years following the onset of her symptoms. A multimodal evaluation of both structural and functional aspects, along with molecular analysis, was performed. We identified a rare *de novo* missense variant in the *IMPDH1* gene (NM_000883.4:c.730A > C, p.Thr244Pro), which had been previously reported in one patient. Threonine 244 is located at the interface between the catalytic domain and the regulatory Bateman domain. This region is crucial for GDP/GTP nucleotide binding and mediates feedback regulation, putatively influencing allosteric regulation. The SD-OCT imaging revealed a symmetrically preserved ellipsoid zone in the central retina, alongside noticeable neuroretinal layer loss beyond the temporal vascular arcades. Functional examinations showed normal BCVA, microperimetric macular thresholds, and color vision thresholds in both eyes. Multifocal ERG was slightly reduced, and full-field ERGs were affected in both dark- and light-adapted responses, consistent with the peripheral sensitivity loss in the 80° automated visual field. No significant changes were observed throughout the follow-up period.

**Conclusions:**

The *IMPDH1* p.Thr244Pro variant was associated with preserved central retina structure and function with stable peripheral dysfunction in the fourth decade of life, suggesting a relatively stationary disease course that starkly contrasts the rapid progression of retinal dystrophies caused by other *IMPDH1* variants.

## Introduction

Retinitis pigmentosa (RP) is the most prevalent (~ 1:2000–4000) inherited retinal disease (IRD) caused by various monogenic alterations resulting in dysfunctional retinal processing due to rod-cone dystrophy, often leading to progressive and irreversible vision loss [1]. RP is genetically heterogeneous and is caused by variants in more than 100 different genes [2]. The pathogenic variants associated with RP may generate similar visual symptoms despite involving different causative genes, affecting specific photoreceptor mechanisms [3]. A further hallmark of RP phenotypes is the immense interindividual variability in disease onset, severity and progression of visual symptoms [4]. Therefore, a detailed exploration of phenotypic peculiarities of certain pathogenic variants is required to optimize the development of gene therapies and the selection of RP candidates undergoing costly upcoming treatments.

The inosine-5-prime-monophosphate dehydrogenase 1 (*IMPDH1,* OMIM: *146,690) gene on chromosome 7 (7q32.1) encodes the regulatory enzyme IMPDH1, which is highly expressed in the inner segments and synaptic terminals of retinal photoreceptors. *IMPDH1* is the causative gene of autosomal dominant retinitis pigmentosa (RP) type 10 (OMIM #180,105), found in approximately 2–4% of patients with RP [3, 5–9]. IMPDH1 operates as a homotetramer to control cell proliferation. The enzyme carries out the rate-limiting reaction in the *de novo* synthesis of guanine nucleotides, which is crucial for maintaining adequate pools of GTP. Since GTP is a precursor for cyclic GMP (cGMP), an essential messenger in phototransduction, IMPDH1 also plays a role in regulating cyclic nucleotide metabolism within photoreceptors. Its alteration can influence cGMP availability, thereby impacting visual signal transduction and the physiological responses of retinal cells to light. The retina has extremely high demands for purine nucleotides, while cGMP plays a crucial role in phototransduction [10, 11].

Since 2002, several pathogenic *IMPDH1* variants have been identified, with Asp226Asn standing out as the most prevalent among the 57–60 disease-causing variants [3, 5–9, 12, 13]. *IMPDH1* gene-associated rod-cone dystrophy (RP10) is characterized by early-onset and severe visual loss. Initial rod alterations are followed by early macular (rod-cone) involvement. Progressive electroretinographic (ERG) alterations in patients with RP10 and the homozygous (*Impdh1*^*−/−*^) mouse model [14] have been reported. In humans, ERGs may become extinguished in the second decade of life [13]. In some *IMPDH1* retinopathies, disease-causing variants disrupt GTP feedback inhibition. Burrell et al. reported that disease-linked *IMPDH1* variants can be classified into two distinct classes: Class I variants are located near the GTP allosteric binding sites and completely abolish GTP-mediated feedback inhibition, whereas Class II variants are situated distally from these allosteric regions and do not alter GTP inhibition. Class I variants are likely to cause dysregulation of nucleotide balance, a condition known to trigger photoreceptor death [10, 15].

The current study reports a rare missense variant (c.730A > C, p.Thr244Pro) in the *IMPDH1* gene associated with a relatively preserved macular function in a 33-year-old female patient.

## Methods

The procedures and data analyses adhered to the tenets of the Declaration of Helsinki and were approved by the Institutional Clinical Research Ethics Committee (SE RKEB 87/2021). Blood samples were collected after signing the Consent Forms.

This clinical case presentation outlines the molecular genetics, ocular findings and visual assessment of a 33-year-old female patient with rod-cone dystrophy. Four consecutive follow-up measurements were performed annually since the onset of the disease. Best corrected visual acuity (BCVA), slit-lamp examination, fundus imaging with autofluorescence, optical coherence tomography (OCT), electroretinography (ERG), Cambridge colour test (CCT), and visual field assessment using automatic Octopus 900 perimeter and MAIA microperimetry were carried out in the Department of Ophthalmology at the Semmelweis University in Budapest (Hungary). Molecular genetic analysis was performed at the Institute of Molecular and Clinical Ophthalmology Basel (IOB, Switzerland), and the segregation analysis and paternity test were performed at the Nephrogenetic Laboratory of the Pediatric Centre, Semmelweis University, Budapest, Hungary.

Blood samples were obtained from the patient and her unaffected mother. Genomic DNA was extracted from peripheral blood leukocytes using conventional methods. The DNA sample from the deceased father was extracted from a formalin-fixed, paraffin-embedded tumor sample for segregation analysis. WES analysis of the female patient was performed using the Twist Human Core Exome Capture Kit (Twist Bioscience) and a NovaSeq 6000 instrument (Illumina). An in-house developed *in silico* bioinformatic pipeline was applied for read alignment, variant calling, annotation, and filtration [16]. Furthermore, AutoMap was used to identify runs of homozygosity from ES data [17]. Finally, we extracted a variant from the screened variants using the ACMG variant classification guidelines that matched the patient’s phenotype. Validation and segregation analysis were performed by Sanger sequencing. From the tumor sample of the deceased father, a short, 80bp-long sequence could be amplified. The forward primer was extended with a 5’ M13 sequence (TGTAAAACGACGGCCAGT), and the sequencing was performed with the M13 primer to obtain sequences of sufficient quality at the variant level. We used Proteinpaint (https://proteinpaint.stjude.org/) to create the representation of IMPDH1 exons and domains.

## Results

### Ophthalmological evaluation

The age at disease onset was 33 years, and the initial clinical manifestation was bilateral, symmetric visual field impairment. The patient did not report nyctalopia spontaneously, it became evident only after targeted questioning.

**Fig. 1:**
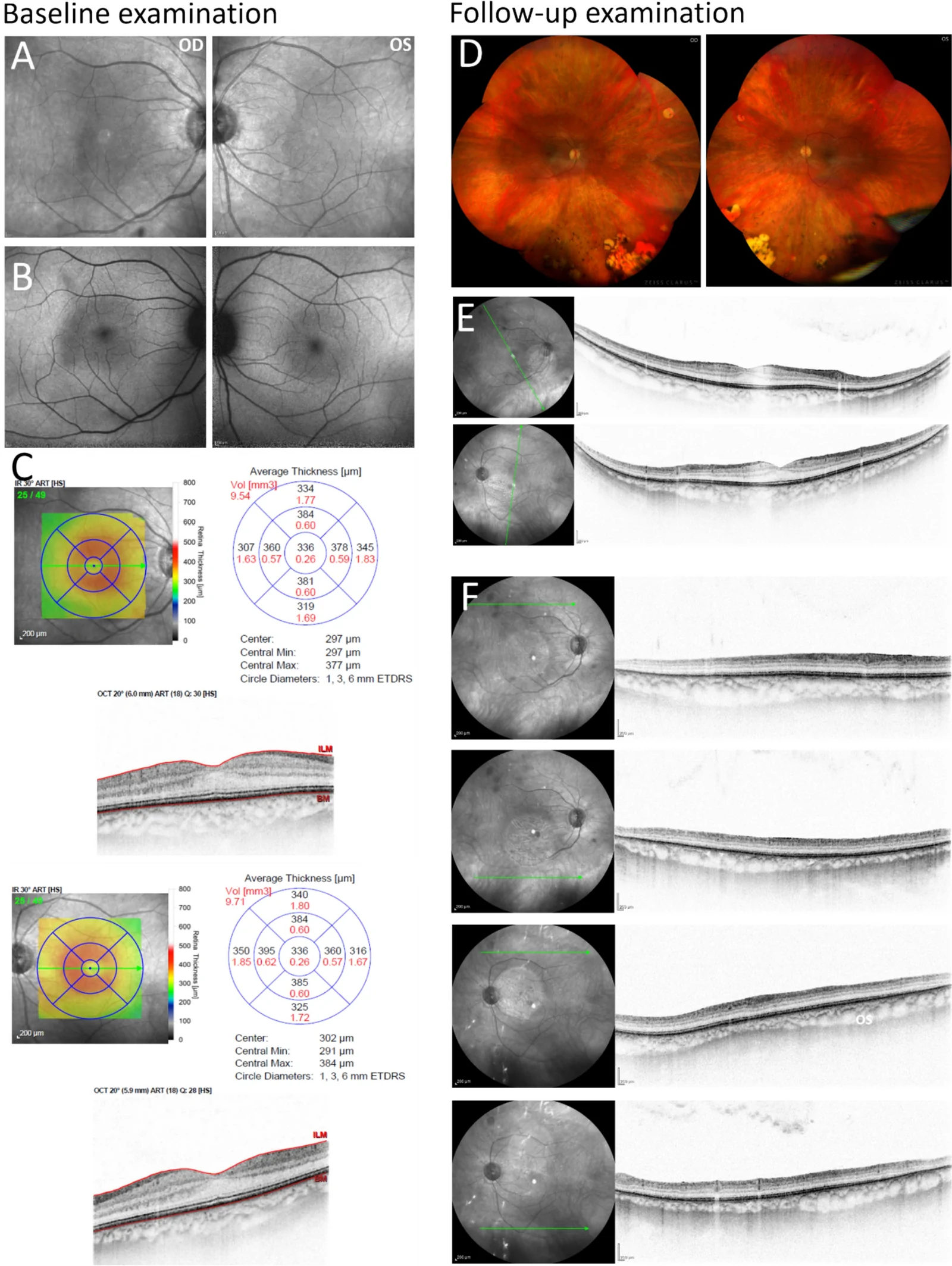
Multimodal retinal imaging at baseline and follow-up visits. Infrared and blue autofluorescence images present a normal appearance (Figs. 1A, 1B, 1E, 1F). The right and left eye scans from the patient showing preserved central foveal ellipsoid zone (Figs. 1C, 1E). Scans from the same patient 4 years later show no significant progression of changes in the ellipsoid zone and all neuroretinal layers, even in the periphe-ral retina (Figs. 1E and 1F). Fundus appearance of the patient with *IMPDH1*-Retinopathy: there were visible pigment deposits and round atrophic spots in peripheral areas of the retina (Fig. 1D, Zeiss Clarus 700, Ultrawide-field image)

Refraction revealed mild astigmatism (SE: −0.50 D) in both eyes after refractive surgery for low-grade myopia (−2.00 dsph in both eyes) six years before the first examination. The best-corrected visual acuity (BCVA) measured with the ETDRS chart was full during the entire period of follow-up (20/20 in both eyes). Biomicroscopy showed an intact anterior segment, along with a clear lens and vitreous body. In the posterior segment, infrared images (Fig. 1A) and fundus autofluorescence appeared normal in the posterior pole (Fig. 1B). SD-OCT imaging indicated an intact neuroretina in the posterior pole, with the ellipsoid zone preserved (Fig. 1C). There were visible pigment deposits and round atrophic spots in peripheral areas of the retina, predominantly in the lower quadrant (Fig. 1D). At the baseline examination, the central macular thickness was 297/302 μm. However, beyond the temporal arch vessels, there was an extensive loss of neuroretinal layers. Follow-up examinations showed no signs of qualitative progression over time (Fig. 1E and 1F).

### Cone- and rod-mediated visual functions

**Fig. 2:**
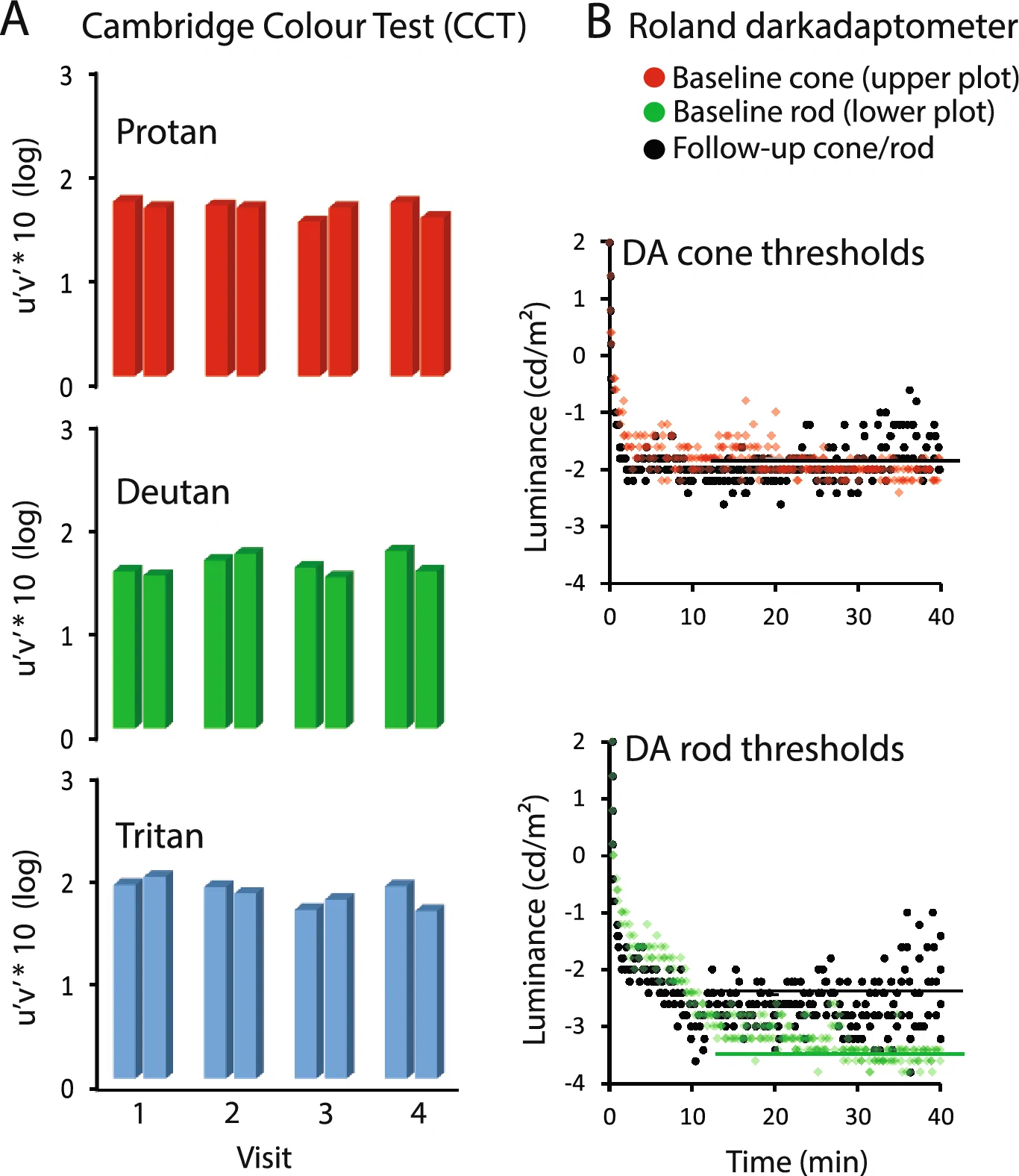
Color vision and dark-adapted thresholds. CCT thresholds to protan, deutan and tritan axes are shown in log units for the right followed by the left eye, at each of the four visits (**A**). Dark-adapted thresholds are shown to cone (red, upper plot) and to rod-dominated (green, lower plot) responses. The average rod threshold was elevated in the follow-up examination (black line) compared to baseline (green line)

Cone–mediated and visual functions were assessed monocularly with the Cambridge Color Test (CCT) and the Roland dark-adaptometer. The CCT is a psychophysical standardized color vision test that allows the determination of color vision thresholds along different directions in the CIE 1976 color space. Visual stimuli consisted of small pseudoisochromatic fields of discs varying in size (between 5.2 and 22.8 arcmin) and in luminance (between 7 and 15 cd/m^2^), forming a mosaic of constant background (u′ = 0.1977, v′ = 0.4689; CIE1976) with the chromaticity of the discs forming a Landolt “C” target which varies during the presentations. The psychophysical procedure generates the color thresholds given as the difference between the chromaticity of the background and the threshold chromaticity in u′ v′* 10^4^. Longitudinal color vision thresholds were below 2 log for protan and deutan and below 2.5 log for tritan, which is considered normal for adults [18]. No significant interocular differences were observed (Fig. 2A).

Dark-adapted visual thresholds were recorded to 625-nm peak wavelength (red light for cone-mediated detection) and 527-nm peak wavelength (green light for rod/cone-mediated detection) flashes of 2-degree diameter presented at around 20 degrees of eccentricity during 40-min dark-adaptation after 5-min bleaching (7000 cd/m^2^). Figure 2B shows the dark-adaptation curves obtained at two time points: baseline and the third follow-up visit. In the first visit, thresholds to either cone-mediated (red = −1.93 log cd/m^2^ upper plot) and rod-dominated (green = −3.50 log cd/m^2^ lower plot) stimulus were normal. The follow-up examination revealed slightly elevated thresholds to rod responses (green = −2.45 log cd/m^2^ black dots and line) compared to the previous examination, while cone threshold (red = −1.85 log cd/m^2^) and cone-rod break (time = 5.01 min) were comparable to the results found in the first examination.

### Macular sensitivity and multifocal ERG (mfERG)

**Fig. 3:**
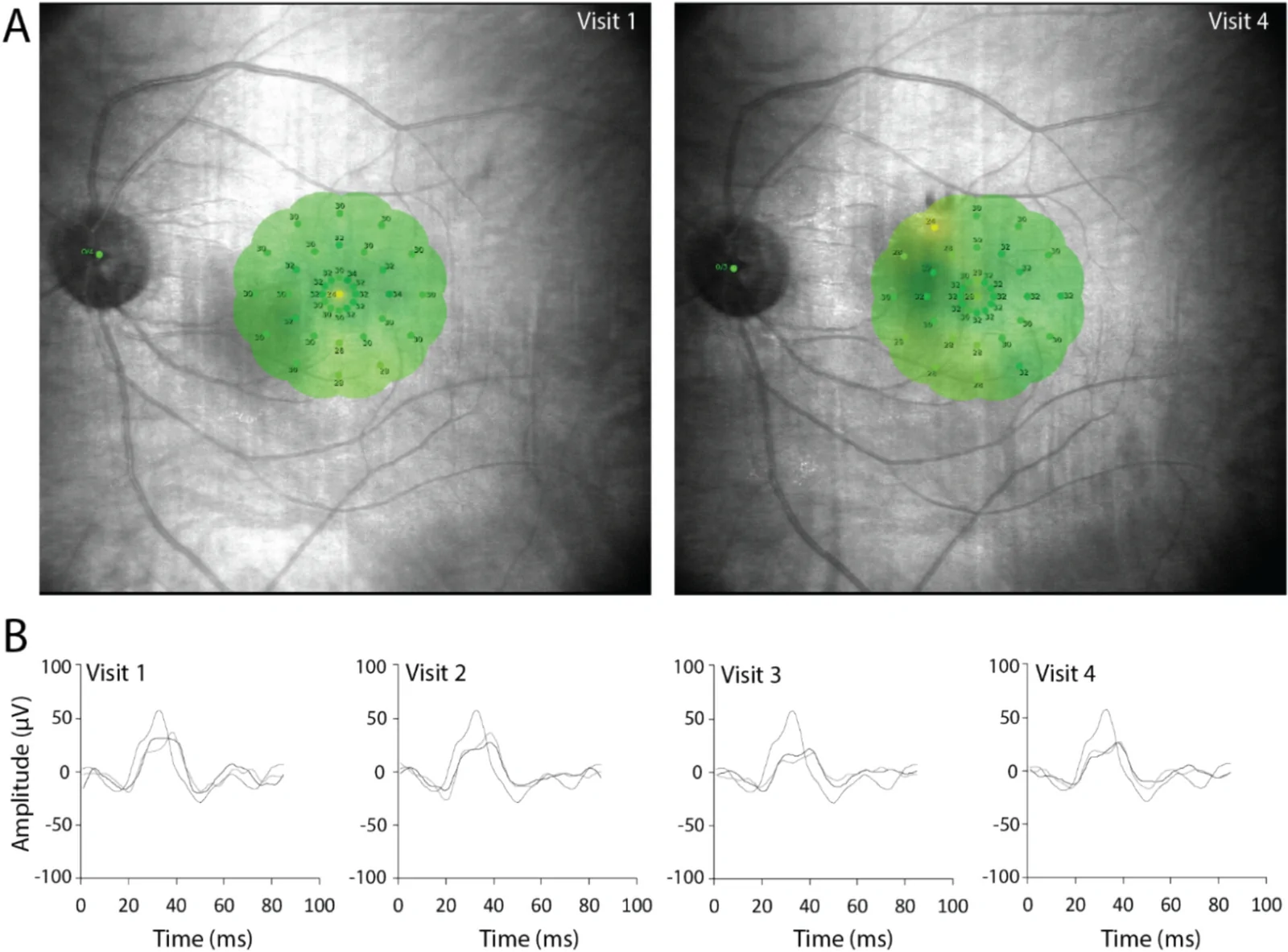
Macular sensitivity and multifocal ERG. Preserved macular thresholds to 10° expert MAIA test obtained in the first and the last visit (**A**). Slightly reduced multifocal ERG amplitudes of the right (black traces) and the left (grey traces) eyes are shown overlapping a control response (thin traces) at each of the four visits (**B**)

The expert protocol of the Macular Integrity Assessment (MAIA; CenterVue, Padova, Italy) microperimetry was applied to record sensitivity at 37 macular points within 10° of the central visual field with simultaneous eye-tracking controlled recordings of the fixation. Standardize (Goldmann III) stimuli were utilized. Normal microperimetric macular thresholds were observed at the baseline examination and during the follow-up period. Figure 3A shows the sensitivity map displaying relatively preserved macular thresholds at the first and the last visit. Average thresholds were 30.9 and 30.8 dB, respectively, during the baseline examination and the last follow-up examination.

Multifocal ERG was recorded with the RETiport system (Roland Consult, Germany) following ISCEV standards [19]. Briefly, the visual stimulus was 61 black and white hexagons covering ~ 50° of the central retina displayed on LCD monitor. Recordings were performed with fully dilated pupils, placing ERGjet electrodes on the corneal surface and reference and ground electrodes (gold cup) attached to the ipsilateral temple and forehead, respectively. Figure 3B shows a trace (average of 61 responses) of a representative control and the traces obtained at the baseline examination (visit 1) and the three consecutive examinations for both eyes, showing relatively stable and slightly reduced amplitudes and a delayed implicit time of the mfERG positive peak. No major interocular variations were observed.

### Peripheral dysfunction

**Fig. 4:**
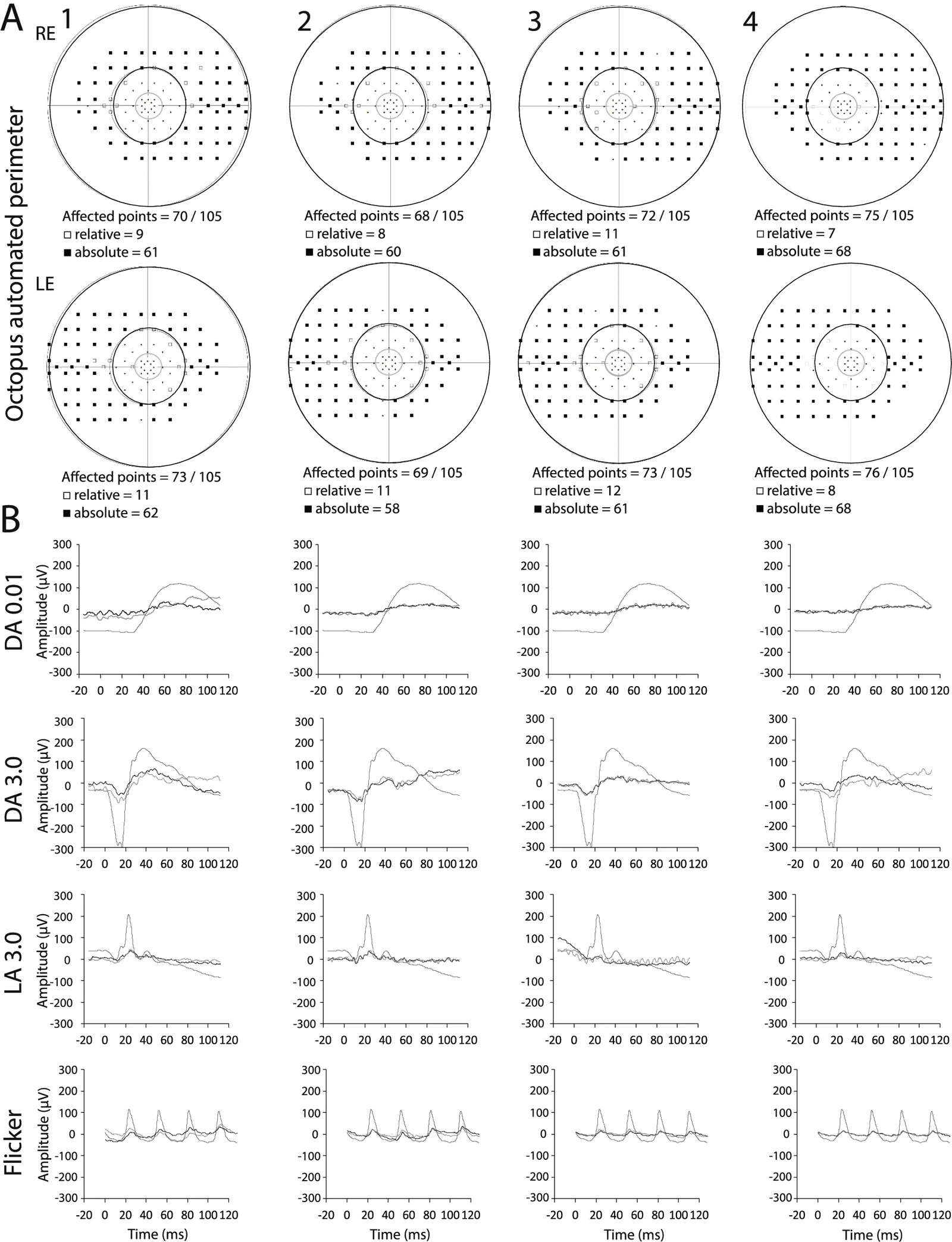
Visual field and full-field ERG. Octopus automated perimeter to 80° standard Test for both eyes at the baseline and follow-up visits (**A**) and the respective dark-adapted (DA) and light-adapted (LA) Full-field ERG recordings of the right (black traces) and the left (grey traces) eyes compared to control responses (thin traces) for each of the four visits (**B**)

Visual field was assessed with the 80° standard 2-Level-Test of the Octopus automated perimeter (HAAG-STREIT, Köniz, Switzerland), recording 105 central and peripheral sensitivity points with Goldmann III standardized stimuli. Figure 4A shows affected areas beyond 30° of the central visual field (large black circles), resulting in absolute scotoma for the majority of the points. In contrast, sensitivity within the 30° of the central visual field (small black circle) showed only a few points with relative defect, while all points within the central 10° (grey circles) showed normal sensitivity. Visual field defects did not change significantly throughout the follow-ups, with no interocular asymmetries.

Full-field ERGs were recorded from both eyes following ISCEV standards [20]. Briefly, the patient was dark-adapted for 30 min. Flash stimuli of 0.01 cd.s/m^2^ (weak flash), 3.0 cd.s/m^2^ (standard flash) and 10 cd.s/m^2^ (strong flash) were presented in sequence with an inter-stimulus time of 2, 10, and 20 s, respectively. Subsequently, the patient underwent light-adaptation to a background of 30 cd/m^2^ for 10 min. Then ERG responses to 3.0 cd.s/m^2^ flashes (standard flash) were recorded with an inter-stimulus time of 0.5 s, followed by a 30Hz-flicker ERG recording. Equipment and electrodes were described above for the multifocal ERG recordings. Reduced, but recordable ERG waveforms were observed at the baseline examination and during the follow-up period. Dark-adapted and light-adapted responses were stable (Fig. 4B).

### *Molecular analysis and in silico protein modelling*

**Fig. 5:**
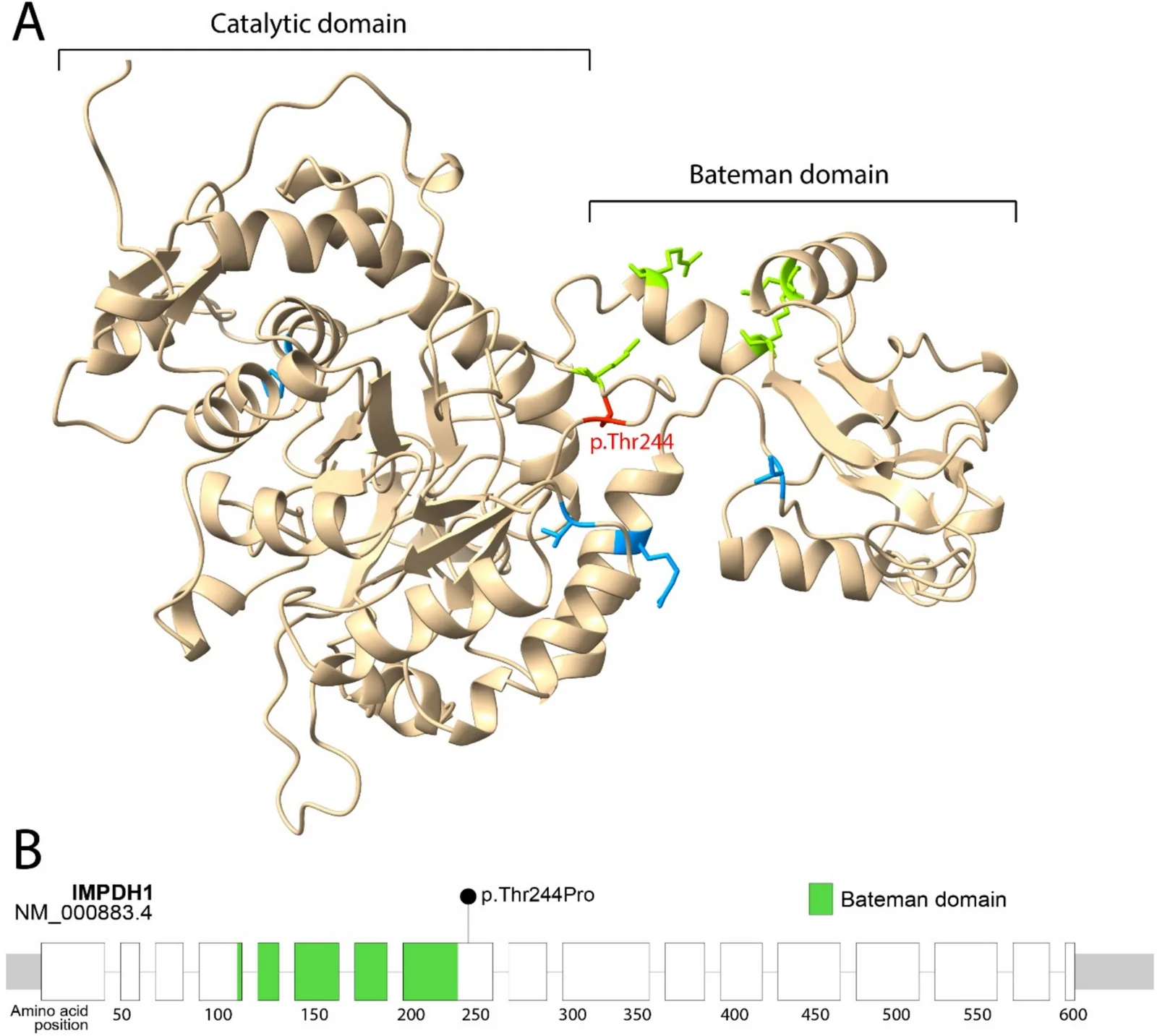
3D model of IMPDH1 protein (AlphaFold, P20839) is shown in panel A and the position of p.Thr244Pro along the exons and protein domains of IMPDH1 is shown in panel B. The amino acid residue affected by the variant detected in the patient is indicated in red. Pathogenic variants causing RP and affecting the Bateman domain are colored in green, while those affecting the catalytic domain are represented in blue according to Burrell et al*.* (PMID: 35,013,599) [15]

**Fig. 6:**
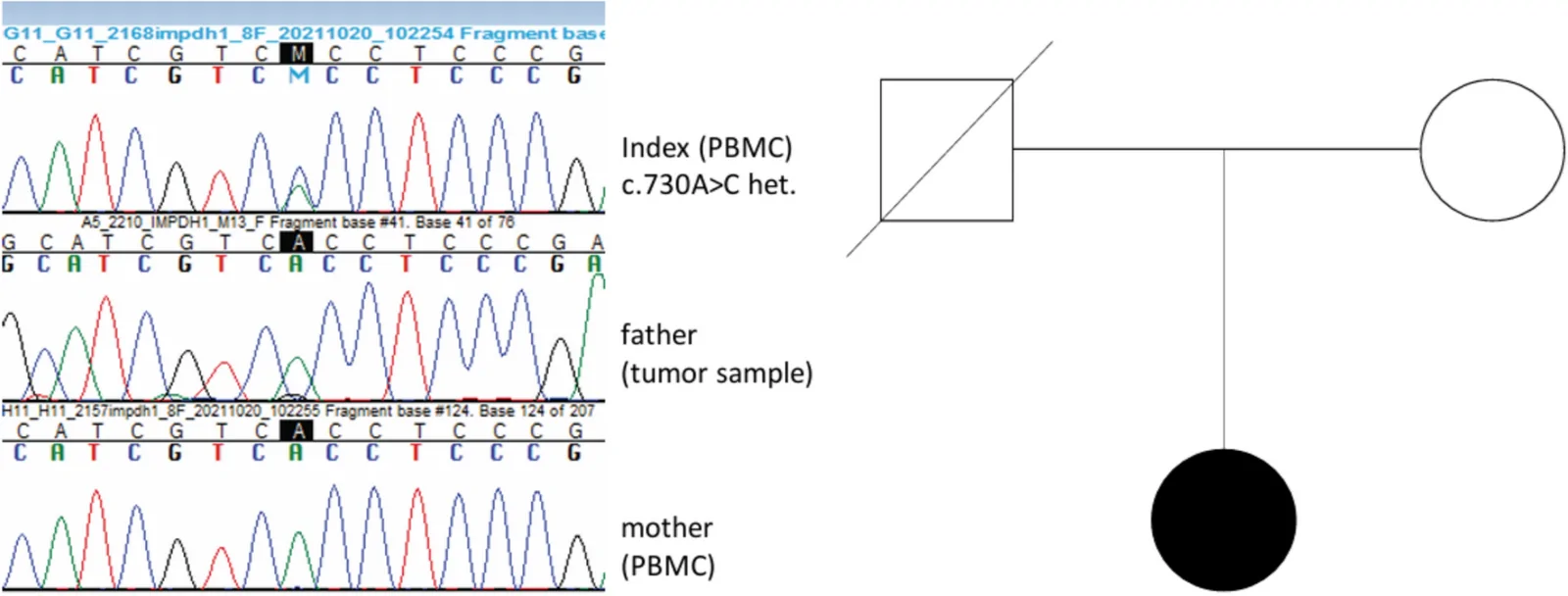
*IMPDH1* exon 8 electropherograms and pedigree. Only DNA extracted from a tumor sample could be obtained from the deceased father. The *IMPDH1* c.730A > C, Thr244Pro variant appeared *de novo*

WES analysis revealed the rare *IMPDH1* c.730A > C, p.(Thr244Pro) variant classified as VUS in ClinVar. This variant is observed in a single heterozygous individual in gnomAD v2.1 and in two individuals in gnomAD v4.1. *In silico* tools predict a deleterious effect (MutScore = 0.879, VEST4 = 0.805), and the affected amino acid is highly conserved across mammals. To date, this variant has been described once in a Polish cohort without further clinical data available [21]. Analysis of the 3D AlphaFold model of IMPDH1 protein (P20839) revealed that the variant is located at the boundary of the Bateman domain and of the catalytic domain. The substitution of threonine with proline is very likely detrimental to the structural integrity of this region, as proline introduces a rigid angle that disrupts the local conformation (Fig. 5A). The schematic representation of the *IMPDH1* gene illustrates the exon structure and corresponding protein domains, highlighting the position of the p.Thr244Pro variant within the gene and its encoded protein (Fig. 5B). This representation provides an overview of the variant’s localization in relation to the functional domains. Segregation analysis revealed that the variant was inherited* de novo* with confirmation of paternity and maternity (Fig. 6). According to the ACMG criteria (PMID: 25,741,868), we classified the variant as likely pathogenic with the criteria: PS2, PM2_sup, PP3_sup.

## Discussion

The phenotypic characterization of *IMPDH1* gene-associated rod-cone dystrophy has been reported to cause early-onset retinal degeneration with progressive peripheral and severe central vision loss in humans [5, 6, 13, 14, 22], while in the *impdh1* knock-out mouse model, slowly progressive retinal alteration has been observed [14]. Affected GTP synthesis in photoreceptors during light activation is believed to underlie *IMPDH1* gene-associated RP [23–25].

Multiple pathological variants of the *IMPDH1* gene have been reported to date: p.Thr116Met, p.Asp226Asn, p.Val268Ile, p.Gly324Asp, p.His372Pro, p.Arg224Pro, and p.Asp226Asn [6, 13]. Here, we report longitudinal clinical examinations (four-year follow-up) from a female adult patient showing a mild phenotype, stable peripheral dysfunctions and preserved central vision associated with a heterozygous de novo* IMPDH1* gene missense variant, p.Thr244Pro. The inosine–5–prime–monophosphate dehydrogenase protein consists of two major domains, the catalytic domain and the regulatory Bateman domain. Threonine 244 is located at the boundary of both domains. This regulatory region is essential for GDP/GTP nucleotide binding and mediates feedback inhibition, thereby potentially influencing allosteric control of enzyme activity [24, 26–28].

The reported phenotypic profiles of the *IMPDH1* gene-associated rod-cone dystrophy point to a relatively balanced rod and cone dysfunction [29], which is in accordance with our findings based on the full-field ERG results: responses to dark-adapted (DA, rod-dominated) and light-adapted (LA, cone-driven) standard flash (DA 3.0 and LA 3.0) were similarly affected at the first visit, although DA ERG to weak flash (DA 0.01) was quite attenuated. Importantly, ERG waveforms were comparable between the first and the last examination. Reduced full-field ERG amplitudes were associated with visual field constriction. The absolute scotoma observed in the peripheral portion of the visual field (30°–80°) and the relative scotoma at central concentric areas between 10° and 30° were in line with the photoreceptoral alterations revealed by the multifocal ERG.

Interestingly, we did not observe any dysfunctions in the foveal and parafoveal regions when examinations were limited to the retina’s central area, such as BCVA, microperimetry (10°) and color vision thresholds (5°). Only slight rod threshold elevation and reduced multifocal ERG amplitudes revealed central retina defects, while BCVA and color vision thresholds were preserved entirely.

The phenotypic characteristics of the preserved central vision observed in our patient may be attributed to the location of the de novo variant. Analysis of the 3D AlphaFold model of IMPDH1 protein (P20839) revealed that the variant is located at the boundary of the Bateman domain and the catalytic domain. The substitution of threonine with proline is likely to compromise the structural integrity of this region, as proline introduces a rigid angle that disrupts the local conformation. Typically, RP caused by *IMPDH1* pathogenic variants presents with a rapid progressive visual loss. The current four-year longitudinal follow-up examination showed that this variant can be associated with a relatively stationary form of retinal dystrophy, which might impact disease prognosis and the design of therapeutic approaches to rescue photoreceptor dysfunctions in RP10-associated heterozygous *IMPDH1*.

Similar phenotypes of *IMPDH1* gene–associated retinal dystrophy presenting with preserved central retinal function have been reported in a Swedish family carrying the Asp226Asn variant [30] and in unrelated pedigrees harboring the p.Asp311Asn variant [31]. Schatz et al. (2005) reported on the clinical and electrophysiological phenotype of a Swedish family carrying the *IMPDH1* variant Asp226Asn, which has been classified as likely benign. Their observations align with our findings, demonstrating a preferential loss of rod function relative to cone function, as well as preservation of central visual function. The authors also noted the presence of intraretinal fluid and retinal swelling, which seemed more pronounced in younger affected individuals [30]. Intraretinal fluid as well as swelling were not observed in the present clinical case. The clinical phenotype has been described for another multigenerational family with the p.Asp226Asn variant. The authors observed color vision deficits, elevated dark-adapted thresholds, and cystoid macular oedema linked to reduced BCVA across all ages. Their findings led them to conclude that the p.Asp226Asn variant results in a severe, early-onset, and progressive form of retinal degeneration. These observations differ from our current results for the p.Thr244Pro variant, underscoring notable discrepancies in disease severity and progression among various *IMPDH1* alleles [22].

Previously underreported variants, such as the one reported here (p.Thr244Pro), might be associated with the group of variants displaying preserved GTP regulation [13], which has been shown to have less impact on BCVA [12].

## Conclusions

*IMPDH1* gene-associated rod-cone dystrophy phenotypically causes early-onset, progressive peripheral and severe central vision loss. However, the severity of these symptoms can vary widely depending on the specific variant present. The p.Thr244Pro missense variant may demonstrate a mild and relatively stationary phenotype. Unlike more severe variants, such as p.Asp226Asn, which result in early and progressive visual degeneration, p.Thr244Pro appears associated with stable peripheral dysfunction, highlighting the phenotypic heterogeneity of *IMPDH1* gene-associated rod-cone dystrophy.

## Data Availability

Exome sequencing data cannot be shared due to restrictions related to the protection of personal data, as mandated by Swiss and European law (GDPR).
